# *Mujer Mas Segura* (Safer Women): a combination prevention intervention to reduce sexual and injection risks among female sex workers who inject drugs

**DOI:** 10.1186/1471-2458-12-653

**Published:** 2012-08-14

**Authors:** Alicia Vera, Daniela Abramovitz, Remedios Lozada, Gustavo Martinez, M Gudelia Rangel, Hugo Staines, Thomas L Patterson, Steffanie A Strathdee

**Affiliations:** 1 Department of Medicine, University of California, San Diego, 9500 Gilman Drive # 0507, La Jolla, CA, 92093-0507, USA; 2 Instituto de Servicios de Salud Publica, Secretaria de Salud de Baja California, Calle Circuito de las Misiones Oriente 188, Parque Industrial Las Californias, Mexicali, BC, 2139, Mexico; 3 Salud y desarrollo Comunitario de cuidad Juarez, Ave. Malecon e Ing. M Cardona, No. 788 Zona Centro, Cd Juarez, Chih, 32000, Mexico; 4 Colegio de la Frontera Norte, Baja California, Frontera, Norte México; 5 Universidad Autonoma de Ciudad Juarez, Pedro Rosales de Leon #7510-117 Colonia Las Fuentes, Cd Juarez, Chih, 32500, Mexico; 6 Department of Psychiatry, University of California, San Diego, 9500 Gilman Drive # 0680, La Jolla, CA, 92093-0680, USA; 7 University of California, San Diego, Institute of the Americas, 9400 Gilman Drive MC 0507, La Jolla, CA, 92093-0507, USA

## Abstract

**Background:**

Female sex workers who inject drugs (FSW-IDUs) are at risk of acquiring HIV, sexually transmitted infections (STI) and blood-borne infections through unprotected sex and sharing injection equipment. We conducted a 2×2 factorial randomized controlled trial to evaluate combination interventions to simultaneously reduce sexual and injection risks among FSW-IDUs in Tijuana and Ciudad Juarez, Mexico.

**Methods/design:**

FSW-IDUs ≥18 years reporting sharing injection equipment and unprotected sex with clients within the last month were randomized to one of four conditions based on an a priori randomization schedule, blinding interviewer/counselors to assignment. Due to the extreme vulnerability of this population, we did not include a control group that would deny some women access to preventive information. All women received similar information regardless of group allocation; the difference was in the way the information was delivered and the extent to which women had an interactive role. Each condition was a single 60-minute session, including either an interactive or didactic version of an injection risk intervention and sexual risk intervention. Women underwent interviewer-administered surveys and testing for HIV, syphilis, gonorrhea, *Chlamydia, and Trichomonas* at baseline and quarterly for 12 months. Combined HIV/STI incidence will be the primary outcome. Secondary outcomes are proportionate reductions in sharing of injection equipment and unprotected sex with clients.

**Discussion:**

Of 1,132 women, 548 (48.4%) were excluded (88.9% were ineligible; 11.1% refused to participate or did not return); 584 eligible women enrolled (284 in Tijuana; 300 in Ciudad Juarez). All 584 participants completed the baseline interview, provided biological samples and were randomized to one of the four groups. During follow-up, 17 participants (2.9%) were lost to follow-up, of whom 10 (58.8%) had died, leaving 567 participants for analysis. This study appears to be the first intervention to attempt to simultaneously reduce injection and sexual risk behaviors among FSW-IDUs. The factorial design will permit analysis to determine whether the combination of the two interactive interventions and/or its respective components are effective in reducing injection and/or sexual risks, which will have direct, tangible policy implications for Mexico and potentially other resource-poor countries.

**Trial registration:**

NCT00840658

## Background

Relationships between drug and alcohol use and unprotected sex have been well documented among female sex workers (FSWs) since early in the HIV epidemic [1-3]. In Holland [4], FSWs who reported engaging in sex work while ‘high’ were more likely to report unsafe sex. In the UK [5], FSWs’ drug use was associated with having unprotected sex in exchange for more money. Unfavorable working conditions including low earnings, limited access to condoms and violence from clients or intimate partners can lead some FSWs to acquiesce to demands for unsafe sex [6,7], especially if they are suffering from withdrawal symptoms or rely on a partner for drugs [8]. Some FSWs exchange sex primarily for drugs [2,9], and/or use drugs with clients, which may increase their risk of sharing injection equipment.

Since female sex workers who inject drugs (FSW-IDUs) are at high risk of becoming infected with HIV through unprotected sexual intercourse and sharing injection equipment with intimate partners, clients and peers, this subgroup meets criteria for a ‘bridge’ population that is associated with generalized HIV epidemics [10]. Overlap between FSW and IDU populations is especially high in parts of Southeast and Central Asia [11], Russia [12,13], and Argentina [14] and is a growing concern in some Latin American countries, such as Mexico [15].

Overlap between FSW and IDU populations in Mexico-U.S. border cities is due at least in part to a number of environmental influences. Sex work is quasi-legal in Mexico, and some Mexican cities (e.g., Tijuana, Baja California), maintain *zona rojas* (red zones) where sex work is tolerated among women aged 18 and older, provided that they subject to quarterly HIV/STI testing and are issued a work permit. Other cities such as Ciudad (Cd.) Juarez, Chihuahua tolerate sex work but do not regulate it. Cd. Juarez began gentrifying their *zona roja* district in 2008, leading FSWs to become dispersed. Both cities have experienced high levels of community-based violence associated with warring drug cartels, but this situation has been particularly severe in Cd. Juarez.

The proximity of Tijuana and Ciudad Juarez to the U.S. cities of San Diego, CA and El Paso, TX draws clients and sex tourists from the U.S. and internationally. In an earlier study, two thirds of FSWs in these cities reported having clients from the U.S., and those that did were more likely to engage in more HIV risk behaviors. Although no robust estimates of the number of FSWs exist for either city, there were an estimated 9,000 FSWs in Tijuana [16] and 4000 FSWs in Ciudad Juarez in 2005 [17].

Mexico is one of the most important sources of heroin and methamphetamine entering the U.S. [18] and it is estimated that 70% of all cocaine entering the U.S. passes through Mexico en route from South America. As a consequence, illicit drug use has increased in Tijuana and Cd. Juarez over the past decade as local consumption markets emerged along drug trafficking routes [18]. In Mexico overall, men were 13 times more likely than women to have ever used an illicit drug, but in Tijuana the ratio was 6:1, indicating a high proportion of female drug users. Although no official estimates of the number of IDUs exist in Mexico, there were an estimated 10,000 IDUs in Tijuana in 2005 [19] and about 6,500 ‘heavy heroin users’ in Cd. Juarez [20] in 2001.

Overlapping sex work and drug use along Mexico’s northern border has influenced local HIV epidemics. By the end of 2005, the estimated number of HIV-infected persons in Mexico was 182,000 [21] with HIV prevalence ~0.6% among adults aged 15–49. However, HIV prevalence was nearly double in Baja California, which ranked 2^nd^ in HIV prevalence among Mexico’s 32 states [21].Although Chihuahua ranked 14^th^ in AIDS incidence across Mexico, a high proportion of cases were attributed to injection drug use [21]. These national and state-level HIV/AIDS surveillance statistics mask a burgeoning HIV epidemic in Tijuana and Cd. Juarez, where HIV prevalence among FSWs rose from 2% in 2003 to 8%, and 12% among FSW-IDUs by 2006 [8]. Almost half of FSW-IDUs in these two cities had at least one sexually transmitted infection (STI), such as HIV, active syphilis, gonorrhea or Chlamydia [8]. In Tijuana, HIV prevalence among female IDUs was 10% in 2006 [22]. Many FSWs in Tijuana and Cd. Juarez report using stimulants like methamphetamine or cocaine to help them cope or stay awake [23], which is of concern since injection and non-injection use of these stimulants was independently associated with HIV infection among FSWs in both [23] cities.

In response to rising HIV prevalence among FSWs in Tijuana and Cd. Juarez, members of our research team conducted a two-arm randomized trial to determine whether a theoretically-based intervention using motivational interviewing was successful in increasing condom use among FSWs in these cities. Conducted from 2004–2006, the study found a 40% reduction in combined HIV/STI incidence associated with this intervention, which was called *Mujer Segura* (Safe Women) [17]. The intervention group also reported higher proportions of protected sex acts compared to the control arm. However, compared to FSWs who had never injected drugs, FSW-IDUs improved less and rates of needle sharing did not change. This was not surprising since the *Mujer Segura* intervention did not focus on reducing drug-related risks, nor did it provide skills to negotiate condom use within the context of drug use.

The high overlap between FSW and IDU populations in countries that bear a high or growing burden of HIV infection suggests that interventions that focus only on safer sex or safer injection will be of limited effectiveness. Yet we could identify no interventions that simultaneously address both sexual and injection drug-related risks for FSW-IDUs. Similarly, we could find no published intervention that taught safer sex negotiation skills within the context of ongoing drug use by FSWs, their regular partners or clients.

The protocol herein describes a four-arm factorial randomized control trial called *Mujer Mas Segura* (Safer Women) to simultaneously test the efficacy of two behavioral interventions-- offered in interactive and didactic formats -- aimed at a) increasing condom use in the context of ongoing drug use; and b) decreasing syringe and paraphernalia sharing among FSW-IDUs in Tijuana and Cd. Juarez. We hypothesized that the joint effect of the interactive format of these interventions will generate greater risk reductions compared to the didactic formats of these interventions. Furthermore, we aimed to examine the extent to which theoretically-based components of these interventions (i.e., knowledge, self-efficacy, outcome expectancies, attitudes, and intentions) represent underlying mechanisms of change in primary outcomes (i.e., sexual and injection-related risk reductions).

## Mujer Mas Segura: study design and methods

The study protocol was submitted, reviewed and approved by Institutional Review Boards in the US (University of California, San Diego, [UCSD]), and Mexico (Centro Nacional para la Prevencion de VIH/SIDA [CENSIDA], Universidad Autonoma de Ciudad Juarez [UACJ] and Hospital General de Tijuana [HGTJ]. In addition, a Data Safety Monitoring Board (DSMB) was formed with experts in the field of public health research, and knowledgeable of the target population of sex workers and injection drug users. To this end, DSMB members reviewed on a regular basis the study’s data on recruitment, retention, adverse events and preliminary estimates of study endpoints (i.e., incidence of HIV and sexually transmitted infections) identifying no issues during the study implementation.

### Study sample

Study recruitment took place between October 28, 2008 through May 31, 2010 in Tijuana and November 15, 2008 through July 30, 2010 in Ciudad Juarez.

#### Inclusion criteria

Participants were required to be: (i) biologically female, (ii) at least 18 years old, (iii) report having exchanged sex for money, drugs, shelter or goods in the last month, (iv) report having injected drugs at least once in the last month, (v) test HIV-negative at baseline, (vi) agree to receive antibiotic treatment for Chlamydia, gonorrhea or syphilis if they tested positive at baseline, (vii) report having had unprotected vaginal or anal sex with male clients at least once during the previous month and (viii) report having shared needles, syringes or other injection paraphernalia (i.e., cookers, cotton, rinse water) at least once within the last month.

#### Exclusion criteria included

(i) consistent use of condoms for vaginal and anal sex with all clients during the previous month; (ii) being <18 years old; (iii) being trans-gendered or biologically male; (iv) testing HIV-positive; (v) having been enrolled in *Mujer Segura*, the pilot study of *Mujer Mas Segura* or another intervention study; (vi) consistently practicing safer sex with clients within the last month, and vii) not having injected with a used syringe or injection equipment (i.e., cotton filter, cookers, rinse water) that had been used by another person in the last month. The age exclusion criterion was imposed because it is illegal for women <18 years old to engage in prostitution in Mexico. Under-age sex workers were referred to partner NGOs for services. Exclusion of transgendered sex workers was imposed because they have different relationship dynamics and sociocultural risk profiles that warranted a potentially different intervention. Pregnancy was not an exclusion criterion; we performed rapid pregnancy tests for women who were unsure if they were pregnant to optimize their STI treatment and referred these women to prenatal care following their visit.

*Description of the Field Teams,* At both sites, the field teams were comprised of outreach workers (2), interviewers (2–3), and field nurses (1–2).

*Outreach workers/promotores*: *Promotore/as* were former or active IDUs, FSWs or FSW-IDUs who were knowledgeable of the target population and were well recognized in the community. On average, there were usually 2 promotore/as to assist with recruitment and follow-up activities in Tijuana and Ciudad Juarez. Most were female but for safety reasons, one male *promotore* at each site assisted female staff during field activities. Male *promotores* would not approach the potential participants directly but would unobtrusively support the female *promotoras* to make sure there were no safety concerns.

*Interviewers/Counselors*: were female college-level graduates such as social workers, psychologists, and health professionals who had > 3 years’ experience working in the community with high risk populations at each site. All of the interviewers were trained on HIV/STI pre- and post-test counseling following national guidelines from the US (US Centers for Disease Control and Prevention) and Mexico (CENSIDA), which were consistent with one another.

*Field nurse*: There were at least 2 nurses available at each site that were responsible for vaginal swab and venipuncture collection. Consistent with the other field team members, field nurses had vast experience and skills necessary to collect biological samples. Field nurses were female and qualified to do HIV/STI pre- and post-test counseling. Field nurses were also responsible for preparing the biological samples for shipping to San Diego.

#### Participant recruitment

We considered sampling strategies to recruit hidden populations, such as respondent driven sampling (RDS) [24,25] and time location sampling (TLS) [24,25]. We elected not to use RDS since a previous study in Tijuana using RDS recruited a low number of female IDUs [26], who tended to have small network chains. TLS was initially attractive, but was eventually ruled out since frequent police crackdowns, army presence and street violence prevented us from maintaining a schedule where we could select venues at random dates and times. Thus, targeted sampling techniques were used, whereby local NGOs already working with FSWs and IDUs were approached to collaborate in the study. Project staff joined local NGO staff during their outreach activities at venues frequented by FSWs and IDUs, which included motels, hotels, brothels, shooting galleries, bars, alleys and street corners in both cities. Potential participants were approached about the study and those who expressed an interest in participating in the study were referred to the project offices or a mobile unit for eligibility screening.

#### Participant screening

A five-minute survey screener was developed to determine if participants met project eligibility criteria. It included questions such as age, whether participants had ever engaged in exchange of sex for money, drugs, goods or shelter or injected drugs and the date they last did so, date of last unprotected vaginal or anal sex act with a client, date they last shared syringes and/or injection paraphernalia (i.e., cotton, cooker, rinse-water), city of residence and intention to move in the next year. A few ‘red herring’ questions (e.g., child care needs, homelessness, border crossing) were also included to prevent women from guessing the eligibility criteria. Staff also checked for track marks to ensure that the participant had injected drugs recently. Women deemed potentially eligible underwent the informed consent process as well as HIV rapid testing. Women who were too inebriated/high to provide informed consent were rescheduled for the next day.

### Data collection

#### Baseline interview

The baseline interview took approximately 40 minutes to complete and was theory-driven and translated into Spanish and back-translated into English by our bilingual, bicultural staff, who also reviewed questions for cultural and linguistic appropriateness. All behavioral measures were administered using computer-assisted personal interviewing (CAPI; NOVA software, MD, USA).

The interview measured socio-demographic and family background (city or town of birth, education, first language, family ties, # of children and dependents, marital status, religiosity or spirituality, sexual abuse, living situation). Financial need was assessed by five items that asked about the participant’s earning from sex work, other sources, number of people who were financial dependents, and the nature of relationship with financial dependents. History, practices and environmental influences regarding substance use included age at first use of alcohol and specific drugs, amount and frequency of alcohol consumed, types of substances used alone and in combination and routes of administration, syringe re-use, receptive and distributive sharing of syringes, injection and non-injection paraphernalia, frequency of injection and syringe sharing, syringe cleaning, needing help injecting, drug use and needle sharing in jail and history of drug treatment. We also collected data on sources of syringes, ease of obtaining sterile syringes, barriers to syringe purchase at pharmacies, shooting gallery attendance, and frequency of arrest and incarceration for charges related to drug possession and paraphernalia. FSWs were also asked to report their use of alcohol and specific drugs preceding and during sex with regular, casual and client partners for the past month.

Sexual behaviors included number and frequency of unprotected vaginal and anal sex with clients, spouse or steady partner and casual male partners in the past month; number of partners who inject drugs, number of female sex partners, and use of the male and/or female condom.

Contextual factors included working conditions: work setting (e.g., brothel, street, bar, motel, hotel), the type of sex worker (e.g., dance hostess, bar maid, street worker), nature of relationship with pimp or manager (if applicable, including degree of financial independence, control over client selection), degree of protection from drunk or aggressive clients, client characteristics, demands for unprotected sex, aggression or violence, amount received for protected vs. unprotected vaginal and anal sex. We also asked about perceived changes in HIV/STI prevention services such as availability of condoms, sterile syringes, HIV testing and medical care, and perceived changes in the environment such as presence of police, army, community violence, and torn down buildings.

Social Influence measures assessed the extent to which social network members engaged in high risk behaviors. Women were asked to think of other FSWs with whom they have the most contact or feel closest to; for each of these individuals, the participant were asked to rate the extent to which this person engages in the following behaviors: sex without condoms; sex with multiple partners without condoms; drug use before or during sex; alcohol use before or during sex; and unsafe injection drug use. Ratings were made on a 4-point scale ranging from 1 (not at all) to 4 (very often). Intrapersonal factors included depressed mood which was assessed using the 20-item Center for Epidemiologic Studies Depression Scale (CES-D) [27]. Self-esteem was assessed using the Rosenberg scale, an 8-item global construct assessing the regard in which one holds oneself [28]. These intrapersonal measures had been used previously in *Mujer Segura*.

### Mechanisms of change variables

#### HIV and HCV knowledge

We utilized a measure of knowledge consisting of 18 items [29], which assessed awareness of the importance of condom use with respect to HIV/STI prevention (e.g., “People who have been infected with HIV quickly show serious signs of illness”). Response categories were True/False.

#### Self-efficacy towards safer injection

This scale was developed in the DUIT intervention study [30] to assess the extent to which IDUs feel they can practice safer injection techniques (e.g., “I can avoid sharing needles even if have had sex without condoms with this person”; “I can avoid sharing needle even if I am in withdrawal *mallila*”). Responses were coded on a 4-point scale (1 = Strongly Disagree to 4 = Strongly Agree).

#### Self-efficacy towards condom use

This 5-item measure was based on a scale developed by Jamner and colleagues [31] and was used in *Mujer Segura.* Participants were asked to indicate the extent to which they are able to use a condom properly with clients, using the same 4-item response categories as above.

#### Peer norms about safer injection

This was a 4-item scale which was previously developed for the DUIT intervention study (alpha = 0.89) [30]. Participants were asked to indicate the extent to which they felt sharing of syringes and injection equipment was normative (e.g., “People I inject with think that sharing cookers, cotton or water should never be shared when they inject”).

#### Outcome expectancies

Participants responded to a 6 item outcome expectancy scale that was developed in *Mujer Segura* which used a 4-point scale ranging from 1 (Strongly Disagree) to 4 (Strongly Agree) [32]. Sample items were “I believe that using condoms will protect me from getting HIV”.

#### Supplemental survey

To minimize respondent burden, a supplemental questionnaire was administered one month after the baseline survey. The following domains were included in the supplemental survey: mobility patterns, including circular migration and migration to U.S. or other locations. We also included Straus’ 39-item measure of intimate partner violence [33] (e.g., ‘Has your partner choked or strangled you’?), that had been piloted in our settings (alpha = 0.87). We also added questions to determine the extent to which women had experienced violence due to conflict over drugs or money with partners, pimps and clients. We assessed power relationship between women and their husband or steady partner using a 4-item scale derived from the Sexual Relationship Power Scale [34] (e.g., “when you and your partner argue, your partner gets his way most of the time”).

#### Randomization

After the screening was completed, participants were assigned to one of four groups based on a randomization schedule that was generated *a priori* by the study statistician. The randomization schedule was not disclosed to the interviewers, ensuring that interviewers were blind to group assignment.

#### Overview of intervention and control conditions

**Figure 1:**
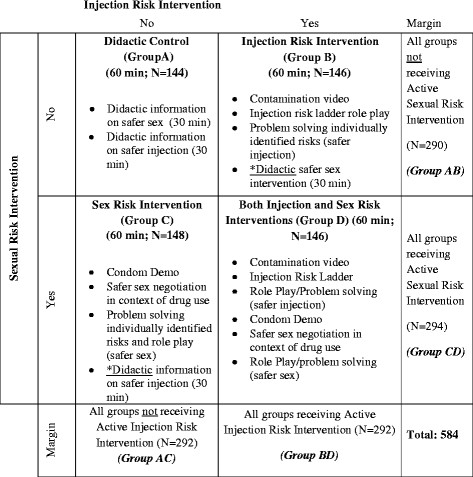
Design of 2×2 factorial trial to simultaneously evaluate injection and sexual risk reduction interventions.

584 HIV-negative women meeting our eligibility criteria (284 in Tijuana and 300 in Ciudad Juarez) were randomized to one of four conditions in a 2×2 factorial design as described below (see Figure 1). We chose a factorial design since it enables simultaneous investigation of two interventions by including all participants in both analyses which is highly cost-efficient. Furthermore, they allow for evaluation of both the separate effects of each intervention and the benefits of receiving both [35]. Because our participants reported engaging in both risky injection drug use and unprotected sex, we felt ethically mandated to provide education on safer injection or safer sex to all women enrolled in the trial. Therefore, each of the four conditions included either an interactive or didactic version of the injection risk intervention and the sexual risk intervention, which were 30 minutes each. Thus, each of the 4 conditions took approximately 60 minutes to complete, which ensured that any observed differences at follow-up were not due to the attention an individual group received. By including a group that received both interactive interventions, it would be possible to determine if FSW-IDUs require active skills training on both sexual and injection risk reduction, or if one of the didactic approaches could achieve similar effect sizes. Experienced interviewers who were indigenous to these communities were trained to deliver this intervention. Each condition is described below. 

#### Group A: Didactic injection risk intervention and didactic sexual risk intervention (60 minutes)

Participants assigned to Group A represented the control condition. They received information on both safer injection and safer sex that was delivered in a lecture-style format and contained information in printed materials available at local health centers. Counselors were instructed not to encourage discussion.

*Didactic injection risk intervention (30 minutes)*. Participants received a didactic presentation where the counselor stressed the importance of using sterile injection equipment to protect against HIV and viral hepatitis and the risks of transmission from sharing injection equipment. The counselor referred women to the local needle exchange and provided the participant with instructions on how to disinfect syringes with bleach in instances where they could not obtain a sterile syringe. This module was designed as to impart education only; no theory-driven active skills building elements oriented towards safer injection were included.

*Didactic sex risk intervention (30 minutes)*. The didactic version of the safer sex intervention was based on a modified version of the CDC guidelines for HIV counseling, testing, and referral [36] and materials from CENSIDA that were used in the control condition of *Mujer Segura*[17]. In this component, there were no theory-driven active skill building elements oriented towards safer sex.

#### Group B: Interactive injection risk intervention and didactic sexual risk intervention (60 minutes)

Women randomized to this group received the interactive injection risk reduction intervention (described below) and the didactic sexual risk intervention (i.e., same module as in Group A).

*Interactive Injection Risk Intervention (30 minutes)*: The injection risk intervention incorporated principles of Motivational Interviewing, Social Cognitive Theory (SCT), and Theory of Reasoned Action (TRA). It was based on components from two multi-site randomized behavioral intervention trials conducted in the U.S. that focused on reducing injection risks among HIV-negative IDUs who were HCV-positive or HCV-negative, respectively; STRIVE *(Study To Reduce Intravenous Exposures)*[37] and DUIT *(Drug User Intervention Trial)*[30]. Both interventions were shown to be highly efficacious in significantly reducing receptive and distributive sharing of injection equipment [38,39], and were listed as evidence based interventions by the U.S. Center for Disease Control and Prevention ( http://www.cdc.gov/hiv/topics/testing/non-healthcare/index.htm)[40]. Details on how components of these interventions were adapted for *Mujer Mas Segura* are described in the Additional file 1.

On a laptop, the counselor then showed the participant a video called “*Una Gota de Sangre*” that was created for *Mujer Mas Segura* (see Additional file 1 for details). The video included a scene whereby a drop of fluorescent dye was to simulate a drop of blood to show participants how their injection equipment can become contaminated and spread HIV or viral hepatitis if injection equipment is shared. When viewed under a black light, the cooker, cotton, water and even the fingers of the person preparing the injection equipment glowed in the dark, showing that contamination from any of these items could occur even if the syringe itself was not shared.

This session also introduced a ‘risk ladder’ whereby participants actively learned how to lower their risk of infection, even if they could not eliminate it entirely. This exercise depicted a ladder with ten rungs and ten cards, each with an accompanying diagram ordered from least risky (i.e. not using drugs) to most risky (i.e., using somebody’s syringe, etc.), and an action item for each that was written on a flash card. The woman was asked to read each card aloud (or if she could not read, the counselor did it for her). The participant was asked to place the card along the ladder, justifying its location depending on the amount of risk each action had for transmitting a blood borne infection. The counselor then used motivational interviewing techniques (e.g., key questions, reflective listening, summarization, affirmation) to elicit information on the woman’s own risky injection behaviors, asking her to reflect on the reasons why she shared injection equipment. Reasons mentioned by project participants included but were not limited to: dependence on someone else for drugs and/or injection equipment, ignorance or fear of where to obtain sterile equipment, and inability to inject herself. Helping the participant understand motivations that underlie sharing injection equipment in different situations was posited to be a prerequisite to behavior change. Using the “decisional balance” approach that is a key tenet of motivational interviewing (MI) [41], the counselor asked the participant to describe the pros and cons of a specific activity—sharing equipment, not using clean works, becoming HIV positive—in order to facilitate the personal realization that these behaviors contained more serious negative than positive outcomes. Since female IDUs in Tijuana and Cd. Juarez reported that their sex partners often influenced their drug use [42], the counselor prompted the participant to verbally propose possible solutions such as (1) obtaining and carrying their own injection equipment; ("works") (2) suggesting that each partner use his or her own "works", or as a last resort, (3) using bleach to disinfect the syringe before using it. As the counselor and participant discussed these underlying motivations, the woman gained insight into her behavior and began to build motivation for change. This was accomplished by eliciting self-motivated reasons for change and enhancing the participant's self-efficacy for change. A role play was then used to help her identify barriers to safer injection using, based on one of three potential injection scenarios (i.e. injecting with a client, sex partner, female friends); each woman chooses a scenario most applicable to her situation. Through the role play, women practiced negotiating skills to empower them not to share their injection equipment and to explain this to those they most commonly injected with.

Finally, women were taught how to disinfect their syringes with bleach, not as a means to replace sterile syringe use, but as a last resort when it was not possible to use sterile syringes (e.g., during incarceration). This provided women with the skills to reduce their risk of infection even in the most undesirable circumstances, which led the women in our pilot study to feel that they really could change their lives. Through these exercises, women were encouraged to verbally state at least one attainable goal to reduce their injection risks (e.g., avoid being high with clients or wait until after sex to use drugs, obtain their own sterile syringe and ‘works’ before using, bleach used syringes). In total, the interactive injection risk intervention was 30 minutes in length.

#### Group C: Interactive sexual risk intervention condition and didactic injection risk intervention condition (60 minutes)

Women randomized to this intervention received the interactive sexual risk reduction intervention (described below), and the didactic injection risk intervention described previously (see Group A).

*Interactive Sexual Risk Intervention (30 minutes)*. The interactive sexual risk intervention was based on components that were included in two interventions that were conducted previously; *Mujer Segura*[17] and *Fastlane*[43], both of which combined the principles of SCT and TRA and used MI to increase condom use. *Mujer Segura* provided FSWs in Tijuana and Cd. Juarez with an interactive intervention aimed at improving their ability to negotiate condom use with clients, but did not take into account the effects of substance use by either partner. *Fastlane* provided heterosexual, HIV-negative women and men with skills to increase condom use in the context of their drug use, but was a multi-session intervention. Thus, the *Mujer Segura* intervention was adapted to incorporate strategies for negotiating condom use within the context of their own, or their partner’s substance use, and was extensively piloted to ensure that it was deemed appropriate for FSW-IDUs in our cultural context.

First, the counselor asked the participant questions about condom use and drug or alcohol use during sex, including her perceived need to change, perceived possibility of change, self-efficacy for change, and stated intentions to change, working to increase her awareness of unsafe sex and associated risks (e.g., HIV, STIs, pregnancy). MI techniques were used to elicit information on her current situation and motivations to help her see her situation clearly and accurately. Based on our pilot work, the lure of more money and drugs is a major reason for not using condoms with clients, whereas power differentials, fear and dependence on her partner for drugs were possible reasons for not using condoms with regular or casual partners. Counselors used the “decisional balance” approach to help the participant articulate that, in most cases, reasons for using condoms strongly outweighed reasons for not doing so. For example, the majority of participants in our study were engaged in sex work to support their children, for whom they wanted to stay healthy. Participants who felt that they could not abstain from drug use were counseled to recognize that by avoiding being high during sexual transactions, they were in a better position to prevent clients from taking advantage or abusing them, and to successfully negotiate condom use.

Once the balance began to shift in favor of positive change, the next step was to help the participant develop a plan of action that best suited her personal situation. The participant was asked to identify barriers to safer sex which typically included the threat of physical assault or death, rape, loss of clients, and, with loss of income, the emergence of withdrawal symptoms (*‘malilla’)*. The counselor then worked with the participant to problem-solve these barriers, offering suggestions on how to change behavior and illustrating positive outcomes. The participant then actively became involved in problem-solving and encouraged to come up with solutions. She was presented with a menu of safer sex options ranging from using condoms, offering oral sex instead of unprotected vaginal or anal sex, avoiding sex when high on drugs, waiting to use drugs after sex and avoiding violent clients. According to both MI and SCT, belief in one's ability to bring about change is an important motivator of change. It was very important that the participant attributed success to herself. The counselor helped the woman define achievable goals (e.g., always use a condom for vaginal and anal sex with clients or always using new, sterile works when injecting drugs). Once the participant had defined goals and arrived at a plan of action, the participant and counselor engaged in a role-play. Alternate strategies for dealing with the situation were discussed, where the woman was rewarded with positive feedback to promote self-efficacy.

The counselor aimed to strengthen the woman’s commitment to using condoms by exploring ways to make condom use exciting and erotic. She showed the participant how to put a condom on properly, and encouraged the participant to practice putting a condom on and off a penis model while discussing how to keep it erect. As we had observed in *Mujer Segura*, many women did not know how to use a condom properly [32]; most reported experiences where condoms broke or stayed inside the vagina, especially when the woman and/or her partner were high. During our pilot work, FSWs expressed gratitude for the condom demonstration and the information regarding proper condom use that were designed to enhance participants’ attitudes, self-efficacy, outcome expectancies, and intentions to use condoms.

#### Group D: Interactive injection risk intervention and interactive sexual risk intervention (60 minutes)

Women randomized to this group received both the interactive injection risk intervention and the interactive sexual risk intervention, which were described above.

#### Outcome ascertainment

To ascertain HIV status, the “Determine”® rapid antibody test was used (Abbott Pharmaceuticals, Boston, MA). All reactive samples were tested using an HIV-1 enzyme immunoassay and immunofluorescence assay. Syphilis serology used the rapid plasma reagin (RPR) test (Determine™ Syphilis TP; Abbott Pharmaceuticals, Boston, MA). Positive samples were subjected to confirmatory testing using the *Treponema pallidum* particle agglutination assay (TPPA) (Fujirebio, Wilmington, DE, USA). Initially, testing for Gonorrhea and Chlamydia was conducted using a rapid test kit (BioStar® OIA® GC and CHLAMYDIA) and positive samples were confirmed on urine specimens using TMA (Genprobe, San Diego, CA). On March 24, 2009, the CT/GC protocol was modified to requesting urine samples from all project participants to accommodate GC/CT screening using the Genprobe Transcription-Mediated Amplification assay (TMA; San Diego, CA). The change in protocol was prompted by the release of a report from the US Centers for Disease Control and Prevention that questioned the sensitivity of the BioStar rapid GC test [44]. *Trichomonas vaginalis* was detected using the OSOM® Trichomonas Rapid Test, and Bacterial Vaginosis using the OSOM® BVBlue® Test (Genzyme diagnostics, San Diego, CA). The San Diego County Health Department (SDCPHL) conducted all confirmatory tests.

At baseline and quarterly follow-up visits, blood specimens were obtained by venipuncture, centrifuged on site, and split into three aliquots. Two vaginal swabs were obtained by a trained nurse (one for gonorrhea and one for Chlamydia testing). Specimens were labeled with the participant’s unique study ID#, date of birth and collection date. Serum was batched and stored at −20 degrees Celsius in on-site freezers until their transport to San Diego on a weekly basis.

#### Biological sample transport

Every effort was made to conduct confirmatory testing locally; however, due to infrastructure and cost-effectiveness biological samples had to be shipped to San Diego for confirmatory testing. Participant samples collected in Ciudad Juarez were shipped to Tijuana via authorized commercial courier (DHL) en route to their final destination in San Diego. Transport of samples constituted a binational effort with the SDCPHL, the State Health Secretary of Baja California and UCSD. A US Centers for Disease Control and Prevention (CDC) permit for the importation of biological samples was secured for the duration of the study and samples were prepared following municipal, state and international standards that regulate the handling, packing, transporting and delivery of biological specimens in Mexico and the U.S. Cross-border transport of samples followed a weekly schedule and was facilitated by a ‘SENTRI’-card holder issued to SDCPHL or UCSD staff.

#### Pre- and post-test counseling and referrals

After the behavioral interview and before the HIV/STI rapid testing was conducted, pre-test counseling for HIV/STI testing was performed as per CDC and Mexican Health Norm guidelines. Results from the rapid HIV/STI tests were ready within 30 minutes, at which time the results were read by a trained counselor and shared with the participant in a private room. Specimens testing positive were immediately repeated with a second rapid test. Participants with an indeterminate or reactive HIV rapid test were referred to Municipal specialty clinic (CAPASITS) for further expedited follow up due to the delay in the availability of the study’s confirmatory test results. Women who tested HIV-positive at baseline were invited to continue in the study to protect their confidentiality but were not included in outcome analyses.

For those participants testing positive on rapid tests to Chlamydia, Gonorrhea, Trichomonas, bacterial vaginosis and syphilis, we provided treatment on the spot while confirmatory testing was being conducted as appropriate. When the gonorrhea rapid test was taken off the market as noted above, we revised the protocol to provide treatment for Chlamydia and Gonorrhea regardless of test results at baseline. In cases of active syphilis (e.g., titers >=1:8), subjects were eligible under Mexico Ministry of Health guidelines for free Penicillin benzatinic treatment regardless of health insurance status. For Trichomonas and bacterial vaginosis cases, participants were administered metronidazole and clindamycin. For gonorrhea and Chlamydia, a one-time antibiotic treatment appropriate for high-risk groups was administered (i.e., Ciprofloxacin, Ceftriaxone and/or Azithromycin) for all women regardless of their randomization assignment, at baseline and follow-up. Study nurses administered all STI medications and provided referrals to municipal clinics when additional medical attention was warranted. Participants were asked to return within one month to receive STI confirmatory test results.

#### Participant incentives

Enrolled participants received reimbursements ranging from $5 to $25 dollars upon completion of specific study activities as follows: a) Five dollars was provided for completing study screening, STI treatment, and locator visits; b) Twenty-five dollars was provided upon completion of each baseline and quarterly follow-up visit.

#### Follow-up interviews

Follow-up interviews were conducted at 1-, 4-, 8-, and 12-month post-randomization and were interviewer-administered using CAPI. The 1-month follow-up visit was scheduled for all participants regardless of group assignment to receive their confirmatory test results. In addition, all participants underwent the supplemental survey, described above. At quarterly follow-up visits, participants were re-tested for HIV and STIs and underwent follow-up interviews with recall periods that referred to the period since the last interview.

#### Cohort retention

A number of strategies were used to minimize attrition. Participants were asked to update their contact information every 4 months in “locator” forms completed by field staff. These locator visits were staggered in between the quarterly follow up interviews. We also implemented street tracking and posted notices at shelters, clinics and drug treatment programs to serve as a visual reminder in public places. The notices only referenced the name of the project, address and phone number of the offices and encouraged participants to call in. Each month, the field coordinator printed a list of those due for an appointment and for those participants who missed their appointments, staff would use the information on the locator forms to actively reach out to them.

Other follow-up strategies included obtaining permission from our institutional review boards in the U.S. and Mexico and the U.S. Office of Human Research Protections to revise our informed consent forms to enable study staff to follow-up participants who became incarcerated. Approval was granted to allow project staff to post a project notice in the nursing station and/or lobby of the institutions and participants who recognized the project information would sign their name up on a project list to be interviewed inside the institution at a later time. Project staff would contact the Institution contact on a regular basis to check whether any names had been added to the list.

In addition, the municipal and state health authorities in Mexico issued official letters (known as “*Oficios*”) supporting the study and requesting the support of other institutions. We obtained *Oficios* from the State HIV/STI Coordinator, the State Secretary of Health, and the Head of Municipal Health Jurisdiction. The authorities at the local penitentiary and directors of the drug rehabilitation centers were formally contacted and they all honored the *Oficios* from the Health authorities and granted access to study participants housed at their facilities.

Finally, our local NGO partners provided project participants with a picture identification (ID) card that served as an informal personal identification card that was honored by most law enforcement and health authorities. The ID card indicated membership to a health program and did not include any information that would reveal the nature of the study to preserve the participant’s confidentiality. This ID card helped study staff confirm the identity of participants at follow-up.

#### Fidelity

We employed the following measures to ensure the fidelity of our interventions: First, all counselors underwent intensive training. Second, a random 10% of counseling sessions were taped with the participant’s permission and were reviewed by the project director and scored for fidelity to the intervention messages. Immediate corrective action and re-training was undertaken when necessary.

#### Contamination

There were at least two potential sources of contamination between intervention arms: (i) our counselors and (ii) cross-talk between women assigned to different conditions. Rigorous training and fidelity checks helped guard against contamination introduced by our counselors. We have found that between-participant contamination is rare (i.e. <1% in *Mujer Segura*). Nevertheless, at follow-up, we asked women if they talked about safer sex or safer injection with another FSW, and if so what the conversation consisted of. If needed, this information can be used as a covariate in our analyses. Despite training, a potential source of bias could be introduced by staff who were aware of subjects’ group assignment. At follow-up, interviewers remained blind to group assignment.

#### Power calculations

**Figure 2:**
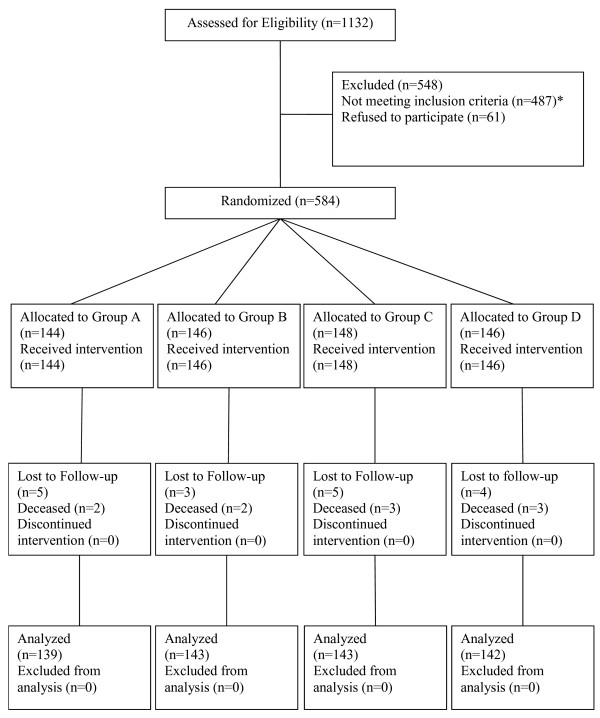
“CONSORT diagram for participants screened, enrolled, randomized and analyzed in Mujer Mas Segura”.

The primary outcome is combined HIV/STI incidence (i.e., confirmed incident cases of HIV, syphilis, gonorrhea, chlamydia, and trichomonas (bacterial vaginosis was not included). Secondary outcomes are reductions in sharing of injection equipment and unprotected sex with clients. We calculated power a priori for our primary outcome (combined HIV/STI incidence), as well as several secondary outcomes (changes in receptive needle sharing, sharing of injection equipment, and condom use with clients). These are described in detail in Additional file 2. These calculations suggested that with an anticipated sample size of 600 participants and a conservative estimate of 20% attrition (N = 480), we would have good power to detect differences across a wide range of possible effect sizes. As shown in Figure 2, 567 women were eligible for analysis due to retention rates that were higher than anticipated (i.e., >90% at each visit at both sites). The study was not originally powered to conduct site-specific analyses.

## Results

A total of 584 eligible FSW-IDUs were recruited into the study, 284 in Tijuana and 300 in Ciudad Juarez. As shown in the CONSORT diagram in Figure 2 and Table 1, a total of 1,132 women were screened for participation, of whom 548 (48.4%) were excluded for the following reasons: 487 (88.9%) were ineligible, and 61 screened as eligible but later did not appear for their interview after they were referred, which can be conservatively interpreted as refusal (11.1%). The main reasons for ineligibility were not having unprotected sex with clients in past month (64%), not injecting drugs in past month (60%), not having exchanged sex for money, drugs, shelter, goods, etc. (45%), and among those who had injected in the past month, not having shared needles or injection paraphernalia (32%). Participants could be disqualified for more than one reason.

**Table 1:** **Reasons for not meeting inclusion criteria for the Mujer Mas Segura study, n = 487**^**†**^

**Disqualification reason**	**Total (n%)**
Did not share needles/syringes/injection paraphernalia in the past month	62(32%)*
Did not have unprotected sex with clients in the past month	311 (64%)
Did not inject drugs in the past month	293 (60%)
Did not exchange sex for money/drugs/shelter/goods in the past month	219 (45%)
HIV-positive	34 (7%)
Plans to move in the next 18 months	63 (13%)
Age <18	6 (1%)
Started sex work < a month ago	3(0.6%)

^†^Numbers do not add to 100% because women could be disqualified for more than one reason; *among those who injected in the past month (n = 194).

Of the 584 eligible women who provided informed consent, all completed the baseline interview, provided biological samples and were randomized at the same visit: 144 were assigned to Group A, 146 to Group B, 148 to Group C and 146 to Group D. During follow-up, a total of 17 participants (2.91%) were lost to follow-up (i.e., did not present for at least one follow-up interview) with death (10/17) being the most common reason (58.8%). All ten deaths were deemed unrelated to study participation by the UC San Diego ethics committee. The remaining 7 participants could not be located. None of the participants who were lost to follow-up withdrew from the study. Therefore, a total of 567 participants were included in outcome analysis as follows: 139 in Group A, 143 in Group B, 143 in Group C and 142 in Group D.

Table 2 provides detailed information about the sociodemographics and baseline characteristics of the 584 participants, overall and by group assignment. Overall, there were few significant differences between the four groups, suggesting that randomization was successful. A marginally higher proportion of participants receiving both interactive intervention conditions (group D) reported obtaining sterile syringes from needle exchange programs at baseline (p = 0.05) whereas a lower proportion reported instances of dividing drugs with a used syringe (p < 0.001) compared to participants in the control group (group A). Otherwise, all four groups were balanced with respect to other demographic and baseline behavioral characteristics, as well as self-reported ease of accessing syringes (p > .05).

**Table 2:** Descriptive statistics of Mujer Mas Segura participants by intervention group at baseline (n = 584)

**Variable**	**Didactic control (n = 144)**	**Injection Risk Intervention (n = 146)**	**Sex Risk Intervention (n = 148)**	**Both, Injection and Sex Risk Intervention (n = 146)**	**Total (n = 584)**	**p**
***Sociodemographics***						
Age (years)	33(27,42)	32(27,40)	34(29,40)	34(28,40)	33(27,40)	.55
# of years of education completed	7(5,9)	6(5,9)	6(5,9)	6(6,9)	6(5,9)	.94
Speaks English	45(31.3%)	43(29.5%)	31(20.9%)	38(26.0%)	157(26.9%)	.20
Has a spouse or steady partner	57(39.6%)	54(37.0%)	48(32.4%)	66(45.2%)	225(38.5%)	.15
Earns average of > = 350 USD**	63(43.8%)	75(51.7%)	69(47.6%)	69(47.3%)	276(47.6%)	.60
# years lived in the city of interview	22(9,31)	21(10,29)	23(10,33)	23(12,34)	22(10,33)	.43
***Injection Drug Use History and Risks***						
Age when first injected drugs	21(17,27)	19(17,24)	19(16,27)	20(18,24)	20(17,26)	.45
Injected > = once per day**	137(95.1%)	140(95.9%)	135(91.2%)	137(93.8%)	549(94.0%)	.35
Obtained syringes from needle exchange**	7(4.9%)	14(9.6%)	19(12.8%)	20(13.8%)	60(10.3%)	.05
Syringe confiscated by police in exchange for not being arrested**	41(28.5%)	39(26.9%)	43(29.3%)	40(27.4%)	163(28.0%)	.97
Often/always injected drugs with a client**	46(31.9%)	41(28.3%)	44(29.7%)	55(37.7%)	186(31.9%)	.33
Receptive needle sharing**	140(97.2%)	140(95.9%)	143(96.6%)	138(95.2%)	561(96.2%)	.81
Divided drugs with used syringe**	101(70.6%)	111(76.6%)	104(70.7%)	84(57.5%)	400(68.8%)	.00
Used a cooker after someone else had used it**	138(96.5%)	140(95.9%)	144(97.3%)	139(95.9%)	561(96.4%)	.90
Used a cotton filter after someone else had used it**	128(88.9%)	127(87.0%)	130(87.8%)	128(88.3%)	513(88.0%)	.97
Sharing rinse water	137(95.8%)	138(94.5%)	139(94.6%)	135(93.1%)	549(94.5%)	.80
Injection Risk Index Score***	3.4( 2.4,4.2)	3.6( 2.4,4.2)	3.6( 2.4,4.0)	3.6( 2.4,4.2)	3.6( 2.4,4.2)	.70
Self-efficacy score for safer drug injection	2.0( 1.8,2.5)	2.1( 2.0,2.7)	2.0( 1.8,2.6)	2.0( 2.0,2.7)	2.0( 1.8,2.7)	.67
Age when began to work regularly as a FSW	19(15,25)	19(16,25)	19(16,24)	20(17,25)	19(16,25)	.22
# Unprotected vaginal/anal sex acts with spouse/steady partner**	8(1,30)	12(4,30)	12(4,30)	8(3,26)	9(3,30)	.53
Income earned from sex (USD)**	885 (286,1883)	975 (440,1740)	1050 (450,1660)	906 (420,1860)	960 (420,1800)	.97
# male clients**	28(10,80)	30(10,84)	38(11,76)	31(14,74)	30(10,80)	.81
# vaginal/anal sex acts with clients**	49(15,109)	57(20,96)	52(20,111)	50(21,100)	51(20,101)	.94
# unprotected vaginal or anal sex acts with clients**	27(6,60)	30(12,57)	33(8,63)	30(8,58)	30(9,60)	.86
Self-efficacy for condom use score	3.0( 2.4,3.0)	3.0( 2.6,3.0)	3.0( 2.6,3.0)	3.0( 2.8,3.0)	3.0( 2.6,3.0)	.17
***Sex Work History and Risks***						
Amount earned per vaginal sex act without condom (USD)	20(13,30)	20(15,30)	20(10,30)	20(15,30)	20(15,30)	.95
Arrested in the past 6 months	68(47.2%)	69(47.6%)	64(43.5%)	61(42.1%)	262(45.1%)	.73
***HIV/STI Services and Laboratory Test Results***						
Ever had an HIV test	74(51.4%)	76(52.1%)	76(51.4%)	66(45.5%)	292(50.1%)	.65
Has seen more access to condoms*	38(26.8%)	40(28.6%)	44(30.8%)	43(30.1%)	165(29.0%)	.88
Has seen more access to sterile syringes*	34(23.9%)	36(25.5%)	42(29.6%)	52(36.1%)	164(28.8%)	.10
Reports ‘easy access’ to sterile syringes	105(75.5%)	117(81.8)	116(81.1)	110(78.0)	448(79.2%)	.54
Tested positive for syphilis	35(24.5%)	36(25.0%)	37(25.3%)	34(23.4%)	142(24.6%)	.98
Syphilis titers > =1:8, among lifetime syphilis cases	10(28.6%)	16(43.2%)	11(29.7%)	12(35.3%)	49(34.3%)	.54
Tested positive for gonorrhea	6(4.2%)	4(2.7%)	1(0.7%)	2(1.4%)	13(2.2%)	.19
Tested positive for Chlamydia	19(13.2%)	22(15.1%)	10( 6.8%)	19(13.0%)	70(12.0%)	.14
Tested positive for trichomonas	40(27.8%)	50(34.2%)	57(38.5%)	49(33.6%)	196(33.6%)	.28
Tested positive for bacterial vaginosis	60(41.7%)	61(41.8%)	62(41.9%)	45(30.8%)	228(39.0%)	.14

*past year; **past month;

*** Calculated based on methods used in the DUIT intervention [38], comprised of the following: receptive needle sharing, sharing a cooker, cotton filter, or rinse water to prepare drugs for injection after someone else had used it, and using a used syringe to divide drugs. The score was constructed by calculating the average between the responses to these five injection risk indicators (1 = never, 2 = sometimes, 3 = about half the time, 4 = often, and 5 = always), with higher scores representing higher risk.

**** Numbers associated with the continuous variables represent Medians and Interquartile Ranges.

### Sociodemographics

Median age, years of education completed and length of residence in the city of interview were 33, 6 and 22 years, respectively.

### Injection drug use history and risks

The median age of first drug injection was 20 years (interquartile range [IQR]: 17–26 years). Regardless of group assignment, most participants reported injecting more than once a day (94%). By definition, all reported either receptive needle sharing or injection with used injection equipment within the last month (e.g., receptive needle sharing: 96.2%; sharing a cooker: 96.4%; sharing a cotton filter: 88.0%; sharing rinse water: 94.5%). An injection risk score was calculated based on methods used in the DUIT intervention [38] study, and was comprised of the following injection risk indicators: receptive needle sharing, sharing a cooker, cotton filter, or rinse water to prepare drugs for injection after someone else had used it, and using a used syringe to divide drugs. The questions regarding these indicators included five item categories (1 = never, 2 = sometimes, 3 = about half the time, 4 = often, and 5 = always). The injection risk score was constructed by calculating the average between the responses to the five injection risk indicators, with higher scores representing higher risk. There were no significant differences in the Injection Risk Index Score across conditions, nor the reported Self-efficacy Score for Safer Drug Injection (Table 2).

### Sex work history and risks

The median age of sex work initiation was 19 years (IQR: 16–25 years). Participants reported having a median of 30 (IQR: 10–80) clients and 30 unprotected vaginal/anal sex acts with clients in the last month (IQR: 9–60). There were no significant differences in the self-efficacy for condom use score across conditions.

### HIV/STI services and lab test results

Fifty percent of the sample reported ever having an HIV test. Slightly less than one-third of the sample reported noticing more access to condoms (29.0%) and sterile syringes (28.8%) in the last year. At baseline, one quarter of the sample tested positive (24.6%) for syphilis, of whom 34.3% had titers >=1:8, which is consistent of active infection. Prevalence of other STIs were: Bacterial Vaginosis: 39%; Trichomonas: 33.6% and Chlamydia: 12%. The prevalence of gonorrhea at baseline was only 2.2%.

### Site differences

Table 3 compares participants in terms of sociodemographics, risk behaviors and HIV/STI results by study site.

**Table 3:** Descriptive statistics of Mujer Mas Segura participants by location: Tijuana (n = 284) versus Ciudad Juarez (n = 300)

**Variable**	**Tijuana (n = 284)**	**Ciudad Juarez (n = 300)**	**Total (n = 584)**	**p**
***Sociodemographics***				
Age (years)	34(28,41)	33(27,39)	33(27,40)	.11
# of years of education completed	8(6,10)	6(4,8)	6(5,9)	<.001
Speaks English	118(41.5%)	39(13.0%)	157(26.9%)	<.001
Has a spouse or steady partner	107(37.7%)	118(39.3%)	225(38.5%)	.68
Earns average of > = 350 USD**	96(34.3%)	180(60.0%)	276(47.6%)	<.001
# years lived in the city of interview	16(8,29)	26(18,34)	22(10,33)	<.001
***Injection Drug Use History and Risks***				
Age when first injected drugs	20(17,24)	20(17,27)	20(17,26)	.47
Injected > = once per day**	275(96.8%)	274(91.3%)	549(94.0%)	.005
Obtained syringes from needle exchange**	26(9.2%)	34(11.3%)	60(10.3%)	.39
Syringe confiscated by police in exchange for not being arrested**	78(27.7%)	85(28.3%)	163(28.0%)	.86
Often/always injected drugs with a client**	134(47.3%)	52(17.3%)	186(31.9%)	<.001
Receptive needle sharing**	271(95.8%)	290(96.7%)	561(96.2%)	.57
Divided drugs with used syringe**	178(63.3%)	222(74.0%)	400(68.8%)	.006
Used a cooker after someone else had used it**	274(97.2%)	287(95.7%)	561(96.4%)	.33
Used a cotton filter after someone else had used it**	256(90.5%)	257(85.7%)	513(88.0%)	.08
Shared rinse water	266(94.7%)	283(94.3%)	549(94.5%)	.86
Injection Risk Index Score	3.6(2.4,4.2)	3.2(2.4,4.0)	3.6(2.4,4.2)	.00
Self-efficacy score for safer drug injection	2.0(2.0,2.8)	2.0(1.8,2.7)	2.0(1.8,2.7)	<.001
***Sex Work History and Risks***				
Age when began to work regularly as a FSW	20(17,25)	19(16,25)	19(16,25)	.02
# Unprotected vaginal/anal sex acts with spouse/steady partner**	9(2,30)	10(4,30)	9(3,30)	.42
Income earned from sex (USD)**	770( 305,1500)	1140( 540,1896)	960( 420,1800)	<.001
# male clients**	15(6,30)	68(30,104)	30(10,80)	<.001
# vaginal/anal sex acts with clients**	34(12,60)	84(40,127)	51(20,101)	<.001
# unprotected vaginal or anal sex acts with clients**	25(5,56)	33(12,65)	30(9,60)	<.001
Self-efficacy for condom use score	3.0( 2.6,3.0)	3.0( 2.6,3.0)	3.0( 2.6,3.0)	.82
Amount earned per vaginal sex act without condom (USD)	25(20,35)	15(10,20)	20(15,30)	<.001
Arrested in the past 6 months	115(40.9%)	147(49.0%)	262(45.1%)	.05
***HIV/STI Services and Laboratory Test Results***				
Ever had an HIV test	144(50.9%)	148(49.3%)	292(50.1%)	.71
Has seen more access to condoms*	113(42.2%)	52(17.3%)	165(29.0%)	<.001
Has seen more access to sterile syringes*	116(43.0%)	48(16.1%)	164(28.8%)	<.001
Reports ‘easy access’ to sterile syringes	238(88.5%)	210(70.7%)	448(79.2%)	<.001
Tested positive for syphilis	46(16.2%)	96(32.7%)	142(24.6%)	<.001
Syphilis titers > =1:8, among lifetime syphilis cases	23(50.0%)	26(26.8%)	49(34.3%)	.006
Tested positive for gonorrhea	5(1.8%)	8(2.7%)	13(2.2%)	.46
Tested positive for Chlamydia	28(9.9%)	42(14.0%)	70(12.0%)	.12
Tested positive for trichomonas	102(35.9%)	94(31.3%)	196(33.6%)	.24
Tested positive for bacterial vaginosis	70(24.6%)	158(52.7%)	228(39.0%)	<.001

*past year; **past month.

*** Calculated based on methods used in the DUIT intervention [38], comprised of the following: receptive needle sharing, sharing a cooker, cotton filter, or rinse water to prepare drugs for injection after someone else had used it, and using a used syringe to divide drugs. The score was constructed by calculating the average between the responses to these five injection risk indicators (1 = never, 2 = sometimes, 3 = about half the time, 4 = often, and 5 = always), with higher scores representing higher risk.

**** Numbers associated with the continuous variables represent Medians and Interquartile Ranges.

### Sociodemographics

Compared to participants in Cd. Juarez, Tijuana participants reported higher levels of formal education (8 vs. 6 years, p = <.001) and a higher proportion spoke English (41.5% vs. 13%, p = <.001). On the other hand, a higher proportion of Cd. Juarez participants reported earning > $350 dollars in last month (60% vs. 34.3%, p = <.001) and had lived in their city of residence for longer durations (median: 26 vs. 16 years, p = <.001), relative to participants in Tijuana.

### Injection drug use history and risks

In the past month, a higher proportion of Tijuana participants reported ‘easy access’ to sterile syringes (88.5% vs. 70.7%, p < .001), having injected drugs at least once a day (96.8% vs. 91.3%, p = .005) and often/always injecting drugs with a client (47.3% vs. 17.3%, p < .001), compared to participants in Cd. Juarez. During the same time period, Cd. Juarez participants were more likely to report having divided drugs with a used syringe (74% vs. 63.3%, p = 0.006). Compared to participants in Cd. Juarez, Tijuana participants scored higher on the Injection Risk Index (p = 0.006) but also scored higher on the self-efficacy scale for Safer Drug Injection (p < .001).

### Sex work history and risks

Compared to Tijuana participants, those in Cd. Juarez reported earning significantly more money from sex work (p < 0.001), had more male clients (p < 0.001), more vaginal/anal sex acts (p < 0.001) and more unprotected vaginal/anal sex acts in the past month (p < 0.001). However, women in Tijuana earned more money for unprotected vaginal sex acts than women in Cd. Juarez (p < .001).

### HIV/STI services and STI test results

Compared to participants in Cd. Juarez, Tijuana participants reported having greater access to condoms (42.2% vs. 17.3%, p < .001) and sterile syringes (43.0% vs. 16.1%, p < .001) in the past year. At baseline, Cd. Juarez participants had higher prevalence of bacterial vaginosis (52.7%. vs. 24.6%, p < .001) and lifetime syphilis (32.7% vs. 16.2%, p < .001) than participants in Tijuana, but of those who had ever had syphilis in their lifetime, a higher proportion of Tijuana participants had syphilis titers >=1:8 relative to Cd. Juarez (50% vs. 26.8%, p = 0.006).

## Discussion

*Mujer Mas Segura* appears to be the first intervention to attempt to simultaneously reduce injection and sexual risk behaviors among sex workers who inject drugs. Due to the extreme vulnerability of this population, we elected not to have a control group that would deny some women access to critically important information that could prevent them from acquiring HIV or other blood-borne or STIs. Instead, all women received similar information regardless of group allocation; the difference was in the *format* the information was delivered and the extent to which women had an interactive role in the way they learned about HIV risk reduction practices. Women randomized to either the interactive injection risk reduction or the interactive sexual risk reduction intervention were encouraged to have an active role in the discussion, stated intentions, role-plays and goal-setting, whereas women receiving the lecture formats of these interventions received only the information in a one-way, didactic format. The 2x2 factorial design should enable us to determine whether the combination of the two interactive interventions and/or its respective components are effective in reducing injection and/or sexual risks, which will have direct, tangible policy implications for Mexico and potentially for other resource-poor countries. Moreover, if either of the lecture formats is sufficient for achieving major risk reductions, this will have important cost implications.

Our interactive intervention modules enabled sex workers to take an active, participatory role to identify their own barriers to safer injection and safer sex and problem-solve with the counselor to suit the context of their lives. By teaching women how to negotiate condom use and inject more safely in the context of their own drug use and that of their clients and intimate partners, we remained sensitive to the reality that some cannot or will not be able to stop using drugs, even if drug treatment is available. Members of our research team were inspired by our experience with the *Fastlane* study of heterosexual, HIV-negative methamphetamine users in the United States, which showed that it was possible for a brief, theoretically based behavioral intervention to increase condom use without attempting to reduce drug use [43].

A unique component of our study was the video we incorporated into the interactive injection risk intervention, which was adapted from a similar video that was used in two multi-site interventions in the U.S. that were highly efficacious [38,39]. While we did not originally intend to feature women in the video who were from the drug user and sex worker communities in northern Mexico, we were surprised that they volunteered to become ‘actors’ in a new video during the piloting phase. The inclusion of FSWs who were active drug injectors as actors who improvised real-world situations they typically encountered was extremely powerful and created a product that other women could relate to. Our approach towards community engagement was consistent with the guidelines developed for working with drug using communities, which recommends “*Nothing About Us Without Us*[45].

Our comparison of sociodemographic and risk behavior variables by intervention group revealed few significant differences, suggesting that randomization was successful in achieving balance across known - and presumably unknown- confounders. On the other hand, we observed important site differences at enrollment that will need to be considered in outcome analysis. This contrasts earlier findings from *Mujer Segura* that suggested that the HIV risks in these cities were similar [32], at least in 2004. At baseline in *Mujer Mas Segura*, women in Tijuana were significantly more likely to report perceived increases in the availability of condoms and sterile syringes and higher proportions reported easy access to syringes compared to Ciudad Juarez. Their experiences are consistent with federal and state-funded initiatives to scale-up HIV prevention and harm reduction services in Tijuana, which began around 2006 [46] in response to evidence that HIV prevalence was increasing, especially among FSWs and female IDUs [16,22,23]. Between 2007 and 2011, the main non-governmental organization (NGO) responsible for syringe exchange in Tijuana reported more than doubling the numbers of syringes exchanged as well as a doubling in the number of ‘prevenkits’ (i.e., kits containing bleach, sterile water, cotton and condoms) (Dr. Remedios Lozada, personal communication, 2012). In contrast, the main NGO responsible for syringe exchange in Ciudad Juarez reported a more modest increase the number of syringes exchanged between 2007 and 2011, and virtually no distribution of prevenkits (personal communication, Maria Elena Ramos, 2012).

Other important differences in the HIV risk environment were apparent between the two cities. Compared to participants in Tijuana, a higher proportion of women in Ciudad Juarez reported seeing changes to the built environment (i.e., torn down buildings), which was likely a result of the city government’s decision to gentrify the *zona roja* district in an attempt to suppress what had been a highly visible sex trade. Anecdotal reports indicate that this effort did not decrease the number of sex workers, but instead dispersed them throughout the city. Women in Ciudad Juarez were also more likely to report seeing an increase in the presence of the federal army compared to Tijuana, which is not unexpected given that mobilization of the federal army was Mexican President Calderon’s response to an extended period of escalating violence in Ciudad Juarez. Finally, women in Ciudad Juarez were more likely to have greater numbers of clients, but earned less per sex act than women in Tijuana.

The stark differences between factors in the HIV risk environment that may influence women’s ability to reduce high risk behaviors or adopt more protective behaviors in these cities represents a unique opportunity to examine a natural experiment. Whether these environmental influences will impact the efficacy of the interactive safer sex intervention, the interactive safer injection intervention, or both is an open question that will need to be explored in site-specific analyses.

## Competing interests

The authors have no competing interests to declare.

## Authors’ contributions

AV, SS, and TP participated in the design of the study and coordination. AV, RL, GM, HS and GR participated in data collection. DA conducted the power calculations, statistical analysis and data management. SS conceived of the study and was the Principal Investigator. All authors read and approved the final manuscript.

## Pre-publication history

The pre-publication history for this paper can be accessed here:

http://www.biomedcentral.com/1471-2458/12/653/prepub

## Supplementary Material

Additional file 1 Appendix A. Click here for file

Additional file 2 Appendix B. Click here for file
