# Supplementary material for: Mujer Mas Segura (Safer Women): a combination prevention intervention to reduce sexual and injection risks among female sex workers who inject drugs
Source: BMC Public Health. 2012 Aug 14;12:653. doi: 10.1186/1471-2458-12-653 (PMC3490986; doi:10.1186/1471-2458-12-653)
Supplement: Additional file 1 — Appendix A. [file 1471-2458-12-653-S1.docx]

**Appendix “A”**

Piloting of the Interactive Injection Risk Intervention:

We piloted a Spanish-translation of a 5 minute video that was developed in the DUIT and STRIVE studies^[^[^30^](#_ENREF_30)^,^ [^47^](#_ENREF_47)^]^ to demonstrate how shared injection equipment can become contaminated and spread HIV or viral hepatitis. The video depicted a simulated injection scene with a female actor in a white lab coat standing in front of a stainless steel table. To a spoon that contained sugar substitute (to simulate powder heroin), the actor added water and a drop of fluorescent dye to simulate a drop of blood. She then proceeded with the simulated process of preparing a syringe for injection. A black light was then illuminated which showed that the cooker, cotton, water and even the actors’ fingers glowed in the dark, showing that contamination from any injection equipment could occur even if the syringe itself was not shared.

*Video modifications:* Project staff conducted focus groups with FSWs in Tijuana and Ciudad Juarez requesting input on the content and cultural appropriateness of the video. The women resonated with the content of the video and were especially impressed with how easily illumination of the dye with black light could translate the concept of contamination. However, several participants commented that the video was too clinical and should be filmed in a more realistic setting that they can relate to (e.g., a home or shooting gallery). Others stated that they would prefer that the video feature Mexican women who were ‘*Tecatas*’ [IDUs] of various ages and social classes representing the diversity of the community. Since black tar heroin is far more common than heroin powder in Mexico, they recommended use of instant coffee to represent heroin when preparing the dose. Candidates provided input on the content of the script and offered suggestions for ‘real life’ scenarios to be included in the video, which included circumstances that can go wrong when trying to inject (i.e., syringe gets clogged up, feeling ‘*malilla*’ (withdrawal symptoms) which heighten the perceived need to borrow syringes, police confiscation of drugs or drug paraphernalia, need to inject after being detained, etc.).

As a result of this feedback, a new video was developed which was referred to as “*Una Gota de Sangre”* (One Drop of Blood). Although a professional company was used to film the video, “casting” sessions were held with female IDUs from Tijuana who were interested in participating as actors. Women who were selected as actors provided written informed consent for their image to be used in the video. The script was drafted by the study investigators but we allowed the women to improvise around the main educational messages to ensure that the video appeared as realistic as possible. The video that was ultimately used in the intervention (with English subtitles added) is available online (insert weblink).

Pre- post assessment of interactive injection risk reduction intervention:

Before producing the new video for *Mujer Mas Segura*, we conducted a pre-post assessment of the interactive injection risk reduction intervention using the Spanish translation of the STRIVE video, adapted versions of the injection risk ladder role play from STRIVE, and role play exercises with 24 FSW-IDUs in Tijuana and Cd. Juarez. Mean age was 33 and mean age at first exchanging sex and injecting drugs were 19 and 22, respectively. In the last month, 83% injected daily, 80% injected with clients and 50% had a regular IDU sex partner. The median duration since last unprotected sex with clients was 2.5 days. Last month, the mean # of unsafe injection and sexual exposures were 11 and 5, respectively. In a pre-post comparison, most subjects improved in knowledge about HIV, HCV and injection risks from contaminated injection equipment. Using a sign test to detect changes in knowledge score, pre-post differences were as follows: “I can tell if my works are contaminated” (p=0.004); “I know how to protect myself from HIV” (p=0.006); I am worried about getting HIV and Hep C” (p=0.06); “Contaminated works can spread HIV” (p=0.002); “Contaminated works can spread Hep C” (p<.001). Although the sample was very small, the findings supported the idea of testing the efficacy of a brief intervention in a larger study of FSW-IDUs.
