# Supplementary material for: Mujer Mas Segura (Safer Women): a combination prevention intervention to reduce sexual and injection risks among female sex workers who inject drugs
Source: BMC Public Health. 2012 Aug 14;12:653. doi: 10.1186/1471-2458-12-653 (PMC3490986; doi:10.1186/1471-2458-12-653)
Supplement: Additional file 2 — Appendix B. [file 1471-2458-12-653-S2.docx]

Statistical Power. Power calculations refer to the Groups defined in Figure 1 and focus on the most conservative contrast (i.e., for Aim 3, cell D is compared with all other cells (defined as Group ABC); intervention effects mentioned below refer to the joint effects of the two interventions). Analyses were not originally planned to be stratified by site, since the Mujer Segura study indicated that there were few significant differences in terms of sociodemographic and risk behaviors between FSWs in Tijuana and Ciudad Juarez.

Power to Detect Change in Receptive Needle Sharing: Using PASS software, we calculated power to detect a significant reduction in receptive needle sharing between Groups D and ABC, conservatively assuming 20% attrition (i.e., N=600×80%=480, sample size ratio in Group D vs. Group ABC=1:3), alpha=0.05 and using logistic regression to detect an intervention effect adjusted for age, duration of injection drug use, and baseline receptive needle sharing (estimated R^2^ for the grouping variable regressed on these three covariates=0.05). Referring to Figure 3, if the proportion of receptive needle sharing in Group ABC is 0.20, 0.30, 0.40 or 0.50, we will have over 80% power to detect an intervention effect if the proportion for Group D is ≤0.09, 0.17, 0.25, 0.35 respectively. Based on the Strive data, among 61 IDUs who had exchanged sex for money in past 3 months, the proportion reporting receptive needle sharing at baseline was 0.62 in the control group and 0.58 in the intervention group (p=0.82). After 6 months, the proportion reporting receptive needle sharing in the control group was 0.33 (95% CI: 0.2–0.5) and the corresponding proportion in the intervention group was 0.07 (95% CI: 0.02–0.23); the difference between these two rates was 0.26 (95%CI: 0.06- 0.44), which is within the range we can readily detect in our *Mujer Mas Segura*. If attrition is less than 20%, power will be even greater.

**Figure 3. Power for Comparing Two Proportions of Receptive Needle Sharing**

| Table 3: Power to detect differences in proportions of injection paraphernalia sharing at a follow-up visit adjusted for covariates including baseline paraphernalia sharing | | | | |
| --- | --- | --- | --- | --- |
|  | Proportion in Group ABC | | | |
| Proportion in Group D | 0.45 | 0.55 | 0.65 | 0.75 |
| 0.20 | 0.999 | 1.0 | 1.0 | 1.0 |
| 0.30 | 0.82 | 0.998 | 1.0 | 1.0 |
| 0.40 | 0.15 | 0.80 | 0.997 | 1.0 |
| 0.50 | 0.15 | 0.15 | 0.81 | 0.998 |

Power to Detect Change in Injection Paraphernalia Sharing: We similarly calculated power to detect a significant reduction in sharing of paraphernalia (i.e., cooker, cotton, and rinse water) between Group D and Group ABC, assuming 20% attrition (i.e., N=480, sample size ratio in Group D vs. Group ABC=1:3), alpha=0.05 and using logistic regression to detect an intervention effect adjusted for age, duration of injection drug use, and baseline injection paraphernalia risk (estimated R^2^ for the grouping variable regressed on these three covariates=0.05). Based on Strive data, among 61 IDUs, the proportion reporting sharing paraphernalia at baseline was 0.85 in the control group and 0.86 in the intervention group (p=1.0). After 6 months, the proportion reporting sharing of paraphernalia in the control group was 0.61 (95% CI: 0.44-0.75) and the corresponding proportion in the intervention group was 0.29 (95% CI: 0.15-0.47). The difference between these two rates was 0.32 (95%CI: 0.07-0.52), which is within the range we can easily detect in *Mujer Mas Segura* (see Table 3, above).

Power to detect change in number and percent of unprotected vaginal sex acts.

| Table 4: Power to detect change from baseline in square root of # unprotected vaginal sex acts | | | | |
| --- | --- | --- | --- | --- |
|  | Change in Group ABC | | | |
| Change in Group D | -1 | -1.1 | -1.2 | -1.3 |
| -1.7 | 0.72 | 0.59 | 0.45 | 0.31 |
| -1.8 | 0.83 | 0.72 | 0.59 | 0.45 |
| -1.9 | 0.91 | 0.83 | 0.72 | 0.59 |
| -2 | 0.95 | 0.91 | 0.83 | 0.72 |

If multiple linear regression analysis is used detect an intervention effect after adjusting for site, time worked as an FSW, duration of injection drug use, and baseline covariates, power calculations below were conducted based on estimates obtained from FSW-IDUs in *Mujer Segura* (using SAS GLMPOWER). Since number of unprotected vaginal sex acts is more appropriate than the proportion of unprotected sex acts for most STI outcomes,^187^ we focused on this as our main sexual behaviors outcome. In *Mujer Segura*, the estimated mean change in square root of number of unprotected vaginal sex acts with clients in the control group was -1.27 (standard error [SE]=0.4) and -1.83 (SE=0.45) in the intervention group. We assumed that the sample size ratio is 1:3 in Group D vs. Group ABC, an attrition rate of 20% and a type I error rate of 0.05. Let ρ denote multiple correlation between all the covariates and a response (change in the square root of the number of unprotected sex acts), root MSE denote the square root of mean square error obtained from an unadjusted linear model that has a response and the group variable only. Table 4 presents power calculations, based on *Mujer Segura* data, to detect an intervention effect for possible combinations of mean changes in our two comparison groups, based on ρ=0.5, and root MSE=3. If we first conservatively assume a decrease of 1 in the square root of unprotected sex acts with clients in Group ABC and 2 in Group D from baseline to a given follow-up visit, and adjust for our four covariates, we estimate our power will be 0.95 (see lower left cell). Greater changes between groups will yield correspondingly higher power.

Power to Detect Changes in Combined HIV/STI incidence: To assess changes in HIV/STI incidence attributable to the interactive sex and injection risk interventions, we generated power curves assuming HIV/STI incidence for Group ABC could range from 0.20 to 0.40 (y-axis), and from 0.01 to 0.30 for Group D (x-axis). We also assumed attrition was 20% (i.e. N=480, sample size ratio in D vs. ABC=1:3) and alpha=0.05. Using subset data from FSW-IDUs enrolled in *Mujer Segura*, logistic regression was used to detect an intervention effect adjusted for site, duration of working as an FSW, duration of injection and report of receptive needle sharing in the past month (estimated R^2^ for the grouping variable (i.e., Group D vs. Group ABC) regressed on these four covariates=0.15). As shown in Figure 4, if HIV/STI incidence in Group ABC is 0.20, we will have ≥80% power to detect an intervention effect if HIV/STI incidence for Group D is ≤0.08; if HIV/STI incidence in Group ABC is 0.30, we will have ≥80% power to detect an intervention effect if HIV/STI incidence for Group D is ≤0.16. Finally, if HIV/STI incidence in Group ABC is 0.40, the study will have at least 80% power to detect an intervention effect if the incidence for Group D is ≤0.24. Based on available data from 71 FSW-IDUs in *Mujer Segura* who did not have HIV/STIs at baseline, the 6-month HIV/STI incidence for the control group was 0.27 (95% CI: 0.16-0.42) and for the intervention group was 0.13 (95% CI: 0.05-0.3). The difference between the two HIV/STI incidence rates was therefore 0.14 (95%CI: -0.06 - 0.31). The observed data, although from a small N, are very close to the numbers used in the above power calculation lending confidence that we have excellent power to measure significant changes in HIV/STI incidence.

**Figure 4. Power for Comparing Two HIV/STI Incidence Rates (Groups ABC vs. D)**
